# Was das Unterhaltungsfernsehen zur Vermittlung von medizinischem Wissen an Studierende und Laien beitragen kann – Sensibilisierung für seltene Erkrankungen

**DOI:** 10.1007/s00103-020-03259-9

**Published:** 2020-12-09

**Authors:** Jürgen R. Schaefer, Eckart von Hirschhausen

**Affiliations:** 1 Dr. Pohl Stiftungsprofessur, Zentrum für unerkannte und seltene Erkrankungen, Universitätsklinikum Marburg, Philipps Universität Marburg, Baldingerstr. 1, 35033 Marburg, Deutschland; 2 Institut für Geschichte der Medizin, Justus-Liebig-Universität Gießen, Gießen, Deutschland

**Keywords:** Dr. House, Dr. von Hirschhausen, Fernsehserie, Medizinische Ausbildung, Gesundheitskompetenz, House M.D., Dr. von Hirschhausen, TV series, Medical education, Health literacy

## Abstract

Menschen mit komplexen und seltenen Erkrankungen haben es in unserem Gesundheitssystem oft schwer. Bis zur Diagnosefindung kann es Jahre dauern und häufig fehlt eine geeignete Therapie. Dabei sind seltene Erkrankungen in der Summe der Patienten alles andere als selten: Allein in Deutschland sind etwa 4 Mio. Menschen betroffen. Dennoch gilt, dass eine seltene Erkrankung oft erst dann entdeckt werden kann, wenn sie bekannt genug ist und die Bevölkerung für ihre Existenz sensibilisiert ist – dies gilt sowohl für Laien als auch die Ärzteschaft. Die eher ungewöhnliche Form der Wissensvermittlung über das Unterhaltungsfernsehen kann einen wichtigen Beitrag zur Verbreitung von medizinischem Wissen und zur Sensibilisierung für medizinische Themen leisten. In konkreten Fällen kann das Unterhaltungsfernsehen so zur Diagnosefindung bei seltenen Erkrankungen beitragen oder Laien zu lebensrettenden Maßnahmen ermutigen, was in diesem Artikel anhand einiger Fallbeispiele verdeutlicht wird.

Serien und Quizshows erreichen sehr viel mehr Zuschauer als klassische Gesundheitssendungen. Auch im Studierendenunterricht haben sie sich als außergewöhnlich wirksam erwiesen. Da die Erzählform das Mitfiebern und Mitraten in den Mittelpunkt stellt; anstelle des reinen Vermittelns von Fakten werden die medizinischen Themen als Gedächtnisinhalte emotional stärker verankert und leichter erinnerlich. Das Unterhaltungsfernsehen bietet somit einen innovativen Ansatz, um die Gesundheitskompetenz der Bevölkerung zu steigern – ein Potenzial, das in Deutschland noch besser genutzt werden könnte.

## Abstract (en)

People with complex and rare diseases often have a difficult time in our health system. It can take years to reach a diagnosis and there is often no suitable therapy. Rare diseases are anything but rare among patients: around 4 million people are affected in Germany alone. Nevertheless, rare diseases can often only be diagnosed when they are well enough known and the population is made aware of their existence—this applies to both laypeople and medical professionals. The rather unusual form of imparting knowledge via entertainment television can make an important contribution to disseminate medical knowledge and to raise awareness of medical topics. In specific cases, entertainment television can help to diagnose rare diseases or encourage laypeople to take lifesaving measures, which we try to illustrate in this paper.

Series and quiz shows reach more viewers than traditional health programs. They have also proven to be exceptionally effective in student teaching. Since the narrative form focuses on cheering and guessing, instead of simply conveying facts, the medical topics become more emotionally anchored memories and easier to recall. Entertainment television thus offers an innovative approach to increase the health literacy of the population—a potential that could be used more intensively in Germany.

## Einleitung

Die moderne Medizin hat gerade in den letzten Jahrzehnten unglaubliche Fortschritte gemacht und ermöglicht die Diagnose von Erkrankungen, die wir vor 40 Jahren noch nicht einmal kannten, und kann Erkrankungen heilen, die vor 20 Jahren noch einem Todesurteil gleichkamen. Hinzu kommt, dass wir durch moderne Computertechnologie und Netzwerke mit Experten konferieren können und das gesamte Wissen der Welt in Sekundenschnelle vor uns haben können, während wir vor 30 Jahren noch mühselige Recherchen in Bibliotheken und mittels Fernleihe durchführen mussten [1, 2].

Aber wie wird das neu gewonnene Wissen verbreitet? Hilfreich für die Recherche nach komplexen und seltenen Erkrankungen sind zum Beispiel spezielle Suchmaschinen, wie FindZebra (https://www.findzebra.com/raredisease). Aber auch Suchmaschinen brauchen zunächst jemanden, der weiß, dass es etwas gibt, nach dem gesucht werden kann, der sensibilisiert ist für die Existenz seltener Erkrankungen. Zudem wird die zunehmende Digitalisierung der Medizin oftmals eher als Bedrohung denn als Chance wahrgenommen [3].

Aufgrund ihres seltenen Vorkommens sind seltene Erkrankungen selbst erfahrenen Ärztinnen und Ärzten oftmals nicht bekannt. Undiagnostizierte oder gar fehldiagnostizierte Patienten mit seltenen oder komplexen Erkrankungen können aber von den medizinischen Fortschritten kaum profitieren, da sie keinen adäquaten Zugang zum Gesundheitssystem finden.

In diesem Beitrag soll auf die Möglichkeiten des Unterhaltungsfernsehens eingegangen werden, sowohl auf die Probleme dieser Menschen hinzuweisen als auch die Diagnostik durch die Erzeugung einer erhöhten Aufmerksamkeit voranzubringen. Im Folgenden wollen wir auf diesen Ansatz mit einigen Beispielen näher eingehen und zeigen, dass er in der Tat Menschenleben retten kann und systematisch mehr genutzt werden sollte.

## Seltene Erkrankungen als Herausforderung für die moderne Medizin

Als „seltene Erkrankung“ gelten in Europa gemäß der Verordnung (EG) Nr. 141/2000 des Europäischen Parlaments und des Rates vom 16.12.1999 all jene Erkrankungen, die eine Prävalenz von nicht mehr als 5 betroffenen Personen auf 10.000 aufweisen und somit nicht häufiger als 1‑mal pro 2000 Personen vorkommen [4–6]. Die Anzahl der seltenen Erkrankungen ist jedoch sehr hoch. Man geht davon aus, dass es zwischen 6000 und 8000 verschiedene seltene Erkrankungen gibt. Eine Zusammenstellung der derzeit bekannten seltenen Erkrankungen ist in der Datenbank Orphanet zu finden [7]. Durch die Vielzahl von seltenen Erkrankungen ist die Gesamtzahl aller Menschen, die von der einen oder anderen seltenen Erkrankung betroffen sind, sehr groß. Man schätzt, dass alleine in Deutschland etwa 4 Mio. Menschen an einer seltenen Erkrankung leiden, also etwa 5 % unserer Bevölkerung und etwa 30 Mio. in der Europäischen Union [8]. In der Tat, würden all die Menschen mit seltenen Erkrankungen in Deutschland eine „Rare-Disease-Partei“ gründen und diese gemeinsam mit ihren Angehörigen und Freunden wählen, säßen sie im Bundestag sowie im Europaparlament. Die Kollegen von Orphanet bringen dies auf den Punkt indem sie sagen: „Rare diseases are rare, but rare disease patients are numerous“ [9]. Doch gäbe es nicht die Allianz Chronischer seltener Erkrankungen (ACHSE e. V.; [10]), die „Eva Luise und Horst Köhler Stiftung“ [11] und zahlreiche engagierte Einzelpersonen, so hätten die „Seltenen“ oftmals weder Stimme noch Lobby.

Selbst überaus erfahrene Ärztinnen und Ärzte können in ihrer gesamten Berufslaufbahn das jeweilige Krankheitsbild nie oder vielleicht nur ein einziges Mal gesehen haben. Für viele Symptomkonstellationen fehlt deshalb der „klinische Blick“, die intuitive Mustererkennung nach Ähnlichkeit und es besteht die Gefahr, dass etwas falsch zugeordnet wird. Dieses Dilemma spüren auch die Patienten und wir müssen uns bemühen, die Aufmerksamkeit sowohl der Ärzteschaft als auch der breiten Bevölkerung auf seltene Erkrankungen zu lenken [12].

Wie wir an den folgenden Beispielen zeigen werden, kann eine Berichterstattung in unterschiedlichen Unterhaltungsmedien dazu führen, dass tatsächlich öfter an seltene Erkrankungen gedacht wird, wenn Menschen von ungewöhnlichen Symptomen betroffen sind, was in der Folge auch häufiger zu den richtigen Diagnosen und Behandlungen führen kann.

## Die Fernsehserie „Dr. House“ in Seminaren für Studierende

Seit 2008 wird am Universitätsklinikum Marburg die TV-Serie „Dr. House“ als „Türöffner“ genutzt, um das Interesse der Medizinstudierenden für seltene Erkrankungen und Diagnosefindungsstrategien zu wecken. Diese Serie (Originaltitel in den USA: „House, M.D.“) dreht sich um den etwas eigenwilligen, fachlich genialen aber menschlich schwierigen Arzt Dr. Gregory House (gespielt von Hugh Laurie). Die Serie besteht aus 8 Staffeln mit insgesamt 177 Episoden und wurde vom Sender RTL im Zeitraum von 2006 bis 2012 im deutschen Fernsehen ausgestrahlt. Sie läuft derzeit weiterhin auf unterschiedlichen Kanälen als Wiederholung und bei diversen Streaminganbietern [13].

In der Serie leitet Dr. House eine Abteilung für Diagnostik und in jeder Episode werden komplexe, oftmals sehr seltene Krankheitsbilder aufgelöst [14]. Dabei ist die fiktive Figur des Dr. House durchaus auch kritisch zu sehen. Er verhält sich ethisch betrachtet oft fragwürdig und sein Fehlverhalten hätte durchaus juristische Konsequenzen. Generell neigen wahrscheinlich alle TV-Formate aus dem Medizinumfeld zu unrealistischen Darstellungen des Klinikalltags, was Witzel et al. an einem Kollektiv chirurgischer Patienten nachweisen konnte [15].

Dennoch – oder gerade deswegen – bietet sich die TV-Serie Dr. House in idealer Weise für ein studentisches Seminar an, das sich mit seltenen Erkrankungen beschäftigt: „Dr. House revisited – oder hätten wir den Patienten in Marburg auch geheilt?“ lautet der Titel des Seminars, in dem mit Medizinstudierenden der klinischen Semester die fiktiven Fälle von Dr. House besprochen werden. Dabei wird auch dessen inakzeptables (und unrealistisches) Verhalten thematisiert. Die Dozierenden bringen zudem eigene reale Fallbeispiele und das tatsächliche Vorgehen mit ein. Die Studierenden werden so für komplexe und seltene Erkrankungen sensibilisiert [16, 17].

Dieses Seminar findet bei den Medizinstudierenden großes Interesse und wurde wissenschaftlich begleitet und ausgewertet [18]. Schon vorher hatte das große Interesse an diesem Seminar dazu geführt, dass am Universitätsklinikum Marburg im Jahr 2013 ein spezielles medizinisches Zentrum für Menschen mit seltenen und unerkannten Erkrankungen (ZusE) gegründet wurde [19–21]. Es ist bemerkenswert, dass ein kleines, aber medial viel beachtetes Seminar für Medizinstudierende die Versorgungsstrukturen einer renommierten, fast 500 Jahre alten Universitätsklinik verändert und selbst bundesweite Wirkung entfacht.

In den letzten 12 Jahren besuchten Hunderte junge Medizinstudierende diese Seminarreihe und wurden so für „seltene Erkrankungen“ sensibilisiert. Interessanterweise melden sich immer wieder ehemalige Seminarteilnehmende, die jetzt als Ärztinnen und Ärzte tätig sind und uns von diagnostischen Erfolgen bei seltenen Erkrankungen berichten. Hier zeigt sich, dass eine TV-Serie wie „Dr. House“ sogar einen nachhaltigen Effekt auf die Patientenversorgung haben kann. Ein recht beeindruckendes Beispiel wird im Folgenden beschrieben.

## Fallbeispiel: Diagnosefindung mithilfe einer TV-Serie

Ein Mitte 50-jähriger Patient mit einer schweren Herzschwäche unklarer Ursache wurde dem Universitätsklinikum Marburg zugewiesen, verbunden mit der Frage, ob eine Herztransplantation notwendig wäre. Eigenartigerweise verlor der Mann seit mehr als einem Jahr zunehmend sein Hör- und Sehvermögen. Zudem hatte er unklares Fieber und eine Schilddrüsenunterfunktion entwickelt – und das alles, wie die Ehefrau bereits beim ersten Gespräch betonte, erst seitdem er eine neue Hüftkopfprothese aus Metall (Kobalt, Chrom und Molybdän) im Austausch für eine zerbrochene Keramikhüftkopfprothese implantiert bekommen habe. Wie sich dann zeigte, lag hierin die Ursache seines komplexen Krankheitsbildes.

Der Patient war gestürzt und seine ursprüngliche Keramikhüftprothese war dabei in zahlreiche kleine Fragmente zersprungen. Ein Orthopäde hatte daraufhin die defekte Keramikprothese entfernt und versucht, die Keramiksplitter aus dem OP-Gebiet herauszuspülen. Um ein erneutes Zerbersten eines Keramikhüftkopfes zu verhindern, hatte er sich entschlossen, einen Metallkopf einzubauen – ein fataler Fehler. Die eingebaute Metallprothese wurde durch im Gelenkspalt verbliebene Keramikreste wie durch Schmirgelpapier zerrieben und so völlig zerstört. Dabei wurden winzige Metallteile ins Blut abgegeben – mit all den oben beschriebenen, verheerenden Folgen einer Kobaltvergiftung.

Dass in Marburg dieser Fall, an dem andere exzellente Kliniker zuvor gescheitert waren, so schnell gelöst werden konnte, war auch das Verdienst der Fernsehserie „Dr. House“. Denn im oben erwähnten Dr.-House-Seminar war wenige Wochen zuvor die Episode mit dem Titel „Spießrutenlauf“ (Staffel 7; Episode 11; Originaltitel: Family Practice) ausführlich besprochen worden, wo genau solch ein Krankheitsbild thematisiert wird. Bei der Veröffentlichung des Falles wurde dies offen zugegeben und bewusst im Titel erwähnt. Die Publikation „Cobalt intoxication diagnosed with the help of Dr House“ löste ein enormes Medieninteresse aus [22]. Das Interesse galt dabei primär der TV-Serie, doch gleichzeitig wurde das Problem fehlerhaft implantierter Hüftkopfprothesen weltweit bekannt [23–26]. Der Beitrag schaffte es laut Altmetric „in the top 5 % of all research outputs scored by Altmetric“ sowie in den „high attention score compared to outputs of the same age (99th percentile)“ [27].

Eine gleichzeitig berichtete ähnliche Fallbeschreibung, die wesentlich umfangreicher und dramatischer war, wurde dagegen kaum beachtet, obwohl sie in einer renommierten Fachzeitschrift publiziert wurde [28, 29]. Dieses Beispiel zeigt, dass die Verknüpfung mit einer populären Serie nicht etwa die Wirkung einer wissenschaftlichen Veröffentlichung schmälert, sondern zu einer viel größeren Wahrnehmung führt.

## Seltene Erkrankungen bekannt gemacht durch Dr. von Hirschhausen

Die ARD-Sendung „Hirschhausens Quiz des Menschen“ [30] vermittelt seit 10 Jahren auf unterhaltsame Weise ernsthafte und wichtige medizinische Themen. Seit der Erstausstrahlung werden pro Sendung zwischen 3,5 Mio. und 4,5 Mio. Fernsehzuschauer erreicht, in der Summe mit den Wiederholungen in den dritten (Regional‑)Programmen bereits mehr als 100 Mio. Menschen. Das Konzept der Fernsehsendung, Medizin unterhaltsam zu präsentieren, beruht auf den Bühnenprogrammen von Dr. Eckart von Hirschhausen, der seit 25 Jahren mit den Mitteln des Kabaretts und der Stand-up-Comedy als Arzt und Wissenschaftsjournalist auf Tour ist und als Sachbuchautor sehr erfolgreich ist.

Der Einfluss von Fernsehsendungen auf Vorsorgeuntersuchungen sowie Arzt-Patienten-Interaktionen wurde bereits wissenschaftlich erforscht [31, 32]. Auch die Wirksamkeit der humorvollen Vermittlung im Liveprogramm von Eckart von Hirschhausen wurde im Kontrollgruppendesign untersucht. Hier ging es speziell um die Bereitschaft zur Organspende [33, 34]. International erfährt die Rolle von Humor in der Wissensvermittlung in Form von Kurzvortragsturnieren (Science-Slams), Serien und Bühnenshows in der Fachliteratur ebenfalls eine neue Wertschätzung [35].

Die tägliche Fernsehdauer ist in Deutschland recht hoch und lag im Jahr 2019 bei rund 211 min – in der Altersgruppe der über 50-Jährigen im Jahresschnitt sogar bei 318 min [36]. Dabei ist der Anteil von spezifischen Gesundheitsformaten sehr viel geringer als der von Unterhaltungssendungen, Arztserien und Krimis. Wie können solche fiktionalen Programme, die auch von weniger gesundheitsbewussten Zuschauern gesehen werden, zur gesundheitlichen Aufklärung beitragen? Bei „Hirschhausens Quiz des Menschen“ vom Westdeutschen Rundfunk ist es gelungen, im Redaktionsteam Unterhaltungsexperten, Ärztinnen und Ärzte sowie Wissenschaftsjournalisten zusammenzubringen. Diese Zusammenarbeit von Wissenschaft und Unterhaltung für eine Quizsendung ist in dieser Form einmalig.

Seltene Erkrankungen werden in der Quizsendung thematisiert, indem zum Beispiel der Protagonist einen Tag lang Menschen in ihrem Alltag begleitet: die gehörlose Tanzlehrerin Kassandra Wedel [37], den Sozialaktivisten Raul Krauthausen mit Osteogenesis imperfecta (Glasknochenkrankheit; [38]) oder Bijan Kaffenberger, ein junger Politiker mit Tourettesyndrom [39]. Dabei steht weniger die Diagnose als die menschliche Seite im Vordergrund, wie auch bei einem Mädchen, dass ein Cochlea-Implantat bekam und jetzt die Welt der Geräusche an und wieder ausstellen kann, je nachdem, ob sie hinhören möchte oder nicht. Trotzdem wird dem Zuschauer indirekt Wissen über die Erkrankungen vermittelt.

Aber auch häufigere Erkrankungen wie Herzstillstand und plötzlicher Herztod werden thematisiert. Hier sind bei der Verbreitung der neuen Regeln zur Wiederbelebung nachweisbare Erfolge erzielt worden. Viele Menschen trauen sich nicht, lebensrettende Maßnahmen wie eine Reanimation durchzuführen. Die Fachgesellschaften haben praktikable Empfehlungen erarbeitet, die auch im Rahmen von „Hirschhausens Quiz des Menschen“ gezeigt wurden: 100 Mal pro Minute drücken – am besten zum gedachten Song „Stayin’ Alive“ von den Bee Gees, da dieser den passenden Rhythmus und die passende Botschaft vermittelt [40]. Eine Frau schrieb, dass sie aufgrund der Sendung ihren Mann erfolgreich wiederbeleben konnte, nachdem er zu Hause einen Herzinfarkt gehabt hatte, und durchhielt bis die Retter kamen. Es gab noch ein halbes Dutzend weiterer E‑Mails von Lebensrettern, die belegen, dass es sich lohnt, im Rahmen von Unterhaltungssendungen lebensrettende Inhalte zu kommunizieren.

In Bezug auf die seltenen Erkrankungen brachte ein Einspielfilm und ein Auftritt bei „Hirschhausens Quiz des Menschen“ mit Schilderung der oben beschriebenen Kobaltvergiftung zahlreiche Anrufer, die aufgrund dieser Berichterstattung erstmals den Ausschluss einer Schwermetallvergiftung nach Auftreten ähnlicher Beschwerden nach dem Einbau eines künstlichen Hüftgelenkes, einer sogenannten Totalendoprothese des Hüftgelenks (Hüft-TEP), anstrebten.

In einer weiteren Sendung wurde ein Fall einer extrem seltenen Lähmung aufgrund einer Kaliumkanalerkrankung vorgestellt. Der Patient litt seit Jahrzehnten an dieser Erkrankung, die ihn zeitweise in eine völlige Bewegungslosigkeit fallen ließ. Letztendlich erkannte der Patient selbst die Ursache seines Leidens, indem er darauf hinwies, dass die Lähmungen mit der Auswahl seiner Speisen zusammenhängen könnten. Er führte ein Ernährungs‑/Befindlichkeitstagebuch und konnte so zeigen, dass die Lähmungen immer dann kamen, wenn er kaliumreiche Speisen zu sich nahm. Ihm wurde eine kaliumarme, eher natriumreiche Kost empfohlen sowie den Kaliumspiegel senkende Medikamente. Fortan waren die immer wiederkehrenden Lähmungsattacken verschwunden [41]. Im Nachgang zu der Sendung gab es zahlreiche Anfragen von Menschen mit ähnlichen Symptomen, die an heimatnahe Zentren für seltene Erkrankungen vermittelt werden konnten [42].

Auch andere seltene Erkrankungen wie Formen der Gehörlosigkeit, Epilepsie, erblicher Magenkrebs und familiärer Darmkrebs waren bereits Thema in einer „Unterhaltungsshow“ von Eckart von Hirschhausen. Der Moderator machte bewusst seine eigene Darmspiegelung öffentlich und vermittelte anhand der Entfernung eines kleinen Polypen einem Millionenpublikum die Bedeutung der Darmkrebsvorsorge.

## Weitere Formate in Unterhaltungsmedien

Es gibt noch weitere sehr gut recherchierte und verständlich gestaltete Formate, die für die Menschen mit seltenen Erkrankungen viel bewirken. Beispielhaft sei hier die Sendung „Abenteuer Diagnose“ vom NDR genannt, aber auch die Rubriken „Die Diagnose“, „Der rätselhafte Patient“ oder „Seltene Erkrankungen“ der Magazine *Stern, Der Spiegel* oder *Focus-Online*. Allen ist gemein, dass sie sehr gut recherchiert sind, von erfahrenen, engagierten Medizinjournalistinnen und -journalisten gestaltet werden und auch komplexe Themen aus dem Bereich der „seltenen Erkrankungen“ verständlich vermitteln können. Dies kann zu einer Sensibilisierung und einem besseren Verständnis dieser Erkrankungen in der Bevölkerung führen, aber auch bei Ärztinnen und Ärzten, wie es sich am Zentrum für unerkannte und seltene Erkrankungen in Marburg schon gezeigt hat.

## Steigerung der Gesundheitskompetenz

Was bislang fehlt, ist eine systematische Verknüpfung der Welt der Medienkanäle, der Redaktionen und Produzenten zurück in die medizinische Fachwelt. Dadurch entstehen immer wieder Situationen, in denen Patientinnen oder Patienten nach einem Krankheitsbild fragen, das ihnen aus den Medien sehr genau bekannt ist, ihre Ärztin oder ihr Arzt jedoch nicht einmal den Krankheitsnamen weiß. Die Verknüpfung findet auch in umgekehrter Richtung kaum statt. So könnten Ärztinnen und Ärzte ihren Patienten gezielt TV-Sendungen in den Mediatheken empfehlen, in denen gute Informationen bereits verständlich aufgearbeitet vorliegen. Es wird leider noch unzureichend auf weiterführende Internetseiten wie das nationale Gesundheitsportal gesund.bund.de verwiesen.

Deutschland liegt in Sachen Gesundheitskompetenz unter dem Durchschnitt der europäischen Länder. Und auch in der Digitalisierung belegen wir einen der letzten Plätze. Im Jahr 2016 sorgte eine Studie mit dem Titel „Gesundheitskompetenz der Bevölkerung in Deutschland“ der Universität Bielefeld für Aufmerksamkeit, nach der sich mehr als die Hälfte der Deutschen im Umgang mit gesundheitsrelevanten Informationen vor Probleme gestellt sehen [43]. Die Qualität der Informationsquellen ist für viele nur schwer oder gar nicht einschätzbar. Nicht nur die Beurteilung, auch das Finden geeigneter und verständlicher Information in den Medien bereitet vielen Menschen Schwierigkeiten. Das gilt auch für gesundheitsbezogene und medizinische Apps. Bislang gibt es kaum Möglichkeiten, sich einen Überblick über diese Angebote zu verschaffen oder deren Qualität einzuschätzen.

Als eine Reaktion entwickelte ein Expertenteam den „Nationalen Aktionsplan Gesundheitskompetenz“ (NAP). Das Strategiepapier sieht unter anderem eine Stärkung der medialen Kommunikationskompetenz der Gesundheitsberufe, die Netzwerkbildung von Medienvertretern, Gesundheitsforschern, Wissenschaftsjournalisten, Kommunikationsfachleuten und Repräsentanten von Patientenorganisationen sowie die freie Verfügbarkeit öffentlich-rechtlicher Beiträge zu Gesundheitsthemen vor [44]. Zudem fordert das Bündnis, dass die Pläne der Bundesregierung zur Schaffung eines steuerfinanzierten „Nationalen Gesundheitsportals“ rasch umgesetzt werden.

## Lernen von den USA

In den USA gibt es eine lange Tradition der Zusammenarbeit von medizinischen Fachgesellschaften mit den Produzenten und Drehbuchautoren von fiktionalen Formaten. Die Serie „Emergency Room“ (ER) entstand vor dem realen Hintergrund der Überlastung der Notaufnahmen in den Vereinigten Staaten. In der Mischung aus temporeicher Schilderung notfallmedizinischer Prozeduren, innovativer Kameraarbeit und politisch-gesellschaftskritischer Themen wurde „ER“ eine der erfolgreichsten Fernsehserien aller Zeiten. Emergency Room hat nachweislich Medizinstudierende inspiriert, sich der Notfallmedizin zuzuwenden, und wird erfolgreich in der Lehre genutzt [45]. Im „Reformstudiengang Medizin“ der Berliner Charité gehört das Vorführen von Sequenzen aus ER zum Block „Notfallmedizin“ des Lehrplanes, um über Notfall- und andere Teamsituationen zu lernen. Anhand der gezeigten Szenen werden Aspekte des Fehlermanagements thematisiert, wie Teamkommunikation in Notfallsituationen, das Einhalten und Übertreten von Kompetenzbereichen oder der Umgang mit schwerwiegenden Fehlern.

Teil des Erfolges von ER war das hohe Niveau der medizinischen Genauigkeit und gezielt eingestreute zentrale Botschaften zur Gesundheitskompetenz. So wussten die Zuschauer, dass George Clooney Faktenwissen erzählt, obwohl er Schauspieler und kein Arzt ist. Ebenso arbeiten Serien wie „Grey’s Anatomy“, „Scrubs“, „Good Doctor“, „Code Black“ oder „House M.D.“ und haben damit durch ihre enorme Reichweite großen Einfluss auf die Wahrnehmung medizinischer Themen in der Bevölkerung [46]. Einige dieser Serien eignen sich sogar, wie bereits beschrieben, als eine Lehrvorlage für die Ausbildung medizinischer Berufe [18].

Von dieser Qualität sind wir in Deutschland leider weit entfernt, obgleich eine beeindruckende „Lernkurve“ sichtbar ist. Wenn aber in einer älteren Folge der Serie „In aller Freundschaft“ zum Beispiel suggeriert wird, ein Patient würde ein Organ direkt an jemanden von der Nachbarstation spenden können, mag das zwar dramaturgisch spannender sein als der europäische Vergabeprozess über Eurotransplant, gleichzeitig werden jedoch alte Ängste und Vorurteile zum Umgang mit Organen genährt statt widerlegt. Bisher werden in Deutschland die Welt der Wissenschaft und der Unterhaltung stark getrennt, ähnlich wie in der Musik die Trennung von „E“ (ernster Musik) und „U“ (Unterhaltungs- und Tanzmusik). Dabei triff der Mensch real weit häufiger die Unterteilung: „interessiert mich“ oder „interessiert mich nicht“. Medizin kann so spannend sein wie ein Krimi. Was könnte alles in einem „Tatort“ nebenbei von den Ermittlern vermittelt werden!

Wie in 2 aktuellen Studien gezeigt werden konnte, wirken sich auch fiktionale Programme auf das Aufsuchen von Vorsorgeuntersuchungen oder die Rollenbilder in Arzt-Patienten-Gesprächen aus [31, 32]. In den USA gibt es von der National Academy of Science das „Science and Entertainment Exchange Program“, in dessen Beirat sich so honorige Wissenschaftler wie Anthony Fauci, Stephen Pinker und Craig Venter mit den wichtigsten Filmproduzenten austauschen [47, 48]. Die Vorteile einer Zusammenarbeit liegen auf der Hand: Für Filmemacher verleihen Wissenschaftsberater ihrer kreativen Vision ein Gefühl von Realismus und Legitimität. Und Wissenschaftler bekommen eine Idee von der Kunst des „Storytellings“, wie man aus realen wissenschaftlichen Geschichten, die jeden Tag um uns herum geschehen, die Inspiration für frische, einzigartige und überzeugende Erzählungen gewinnt.

## Zusammenarbeit von Medizin und Medien in Deutschland

Auch in Deutschland wurde seit der „Schwarzwaldklinik“ (ZDF; 1985 bis 1989) einiges getan, um medizinische Inhalte realistischer darzustellen. Das medizinische Coaching geht aber von einzelnen externen Firmen und Personen aus, deren Kosten als Erstes eingespart werden, wenn das Geld knapp wird. Eine systematische Qualitätssicherung findet nach Wissen der Autoren bisher nicht statt. Medienwelt, Gesundheitsforscher, Wissenschaftsjournalisten, Kommunikationsfachleute und Patientenorganisationen haben noch wenig Berührungspunkte und Austausch. Dabei wäre die Zusammenarbeit sehr fruchtbar: Während die einen die großen Lücken im Gesundheitswissen der Bevölkerung kennen, wissen andere, wie gute Serien, Magazine oder Unterhaltungssendungen gemacht werden, und wiederum andere wissen, wonach Patientinnen und Patienten eigentlich suchen und worauf sie angewiesen sind, um sich im Gesundheitsdschungel zu orientieren. Es ist wichtig, dass diese verschiedenen Welten und Akteure zusammenkommen, sich kennenlernen, voneinander lernen und gemeinsam Ideen entwickeln.

Für die Berichterstattung von Wissenschaftsjournalistinnen und -journalisten ist das von der Klaus Tschira Stiftung (https://www.klaus-tschira-stiftung.de/) und der Wissenschaftspressekonferenz gegründete „Science Media Center Germany“, als eine unabhängige und gemeinwohlorientierte Wissenschaftsredaktion, enorm hilfreich, um durch Einbindung ihres Expertennetzwerkes eine zeitnahe wissenschaftliche Einschätzung zu aktuellen Ereignissen in der Wissenschaft kostenfrei für Redaktionen zur Verfügung zu stellen (https://www.sciencemediacenter.de). Der Service endet aber bislang mit einem Text mit Quellenangaben und O‑Tönen zur Einordnung. Die „Übersetzung“ in Grafiken, Bewegtbilder oder gar in andere reichweitenstarke Formen geschieht dann an anderer Stelle – oder gar nicht.

Die Klaus Tschira Stiftung hat das Nationale Institut für Wissenschaftskommunikation (NaWik) in Karlsruhe gegründet. Gemeinsam mit „Wissenschaft im Dialog“ (WiD) und Frau Prof. Annette Leßmöllmann werden so am Karlsruher Institut für Technologie (KIT) mit der Plattform „wissenschaftskommunikation.de“ innovative Wege der Wissenschaftskommunikation vorangetrieben. Aber die wenigsten dieser löblichen und wichtigen Ansätze kümmern sich bislang um die Einbindung der fiktiven und unterhaltenden Formate. Dabei hat die Medienwirkungsforschung über COVID-19 gezeigt, dass es viele Menschen gibt, die über die üblichen Kanäle nicht zu erreichen und zu überzeugen sind, und Videos mit Verschwörungstheorien und Wissenschaftsfeindlichkeit enorme Resonanz bekommen. Es braucht also dringend neue Wege, die evidenzbasierte und gut erzählte Wissenschaft durch eine institutionalisierte Verbindung mit der Kreativbranche zu stärken. Ein guter erster Schritt könnte eine Tagung sein, wie sie beispielsweise das „Grimme-Institut“ zur Rolle von Behinderten in den Medien veranstaltet hat [49]. Wünschenswert wäre auch eine Stärkung der Wirkungsforschung der verschiedenen „Darreichungsformen“ von Informationen auf verschiedene Zielgruppen, um auf diesem Weg mehr Ärzte, Patienten und Angehörige sowohl über die seltenen als auch über oftmals vernachlässigte häufige Erkrankungen zu informieren. Hier sollten in einem wissenschaftlichen Diskurs Nutzen und Risiken einer engeren Zusammenarbeit von Medien und Medizin ausgearbeitet werden, um so unser Gesundheitswesen in Deutschland zu verbessern.

## References

[1] Müller T, Jerrentrup A, Schäfer JR (2018). Computerunterstützte Diagnosefindung bei seltenen Erkrankungen [Computer-assisted diagnosis of rare diseases]. Internist.

[2] Müller T, Jerrentrup A, Fritsch H-W, Schäfer J (2016). Software zur Unterstützung der Differenzialdiagnose in der Inneren Medizin – Auswirkungen auf die Qualität der Medizin [Software to support differential diagnosis in internal medicine – Impact on the quality of medicine. Klinikarzt.

[3] https://www.bertelsmann-stiftung.de/fileadmin/files/BSt/Publikationen/GrauePublikationen/VV_Studie_Algorithmen.pdf. Zugegriffen: 26. Aug. 2020

[4] https://ec.europa.eu/health/sites/health/files/rare_diseases/docs/2014_rarediseases_implementationreport_en.pdf. Zugegriffen: 26. Aug. 2020

[5] https://www.bundesgesundheitsministerium.de/fileadmin/Dateien/5_Publikationen/Praevention/Berichte/110516_Forschungsbericht_Seltene_Krankheiten.pdf. Zugegriffen: 26. Aug. 2020

[6] https://www.gesundheitsforschung-bmbf.de/de/seltene-erkrankungen-6437.php. Zugegriffen: 26. Aug. 2020

[7] http://www.orpha.net/orphacom/cahiers/docs/DE/Verzeichnis_der_seltenen_Krankheiten_in_alphabetischer_Reihenfolge.pdf. Zugegriffen: 26. Aug. 2020

[8] https://www.bundesgesundheitsministerium.de/themen/praevention/gesundheitsgefahren/seltene-erkrankungen.html. Zugegriffen: 26. Aug. 2020

[9] https://www.orpha.net/consor/cgi-bin/index.php?lng=EN. Zugegriffen: 26. Aug. 2020

[10] https://www.achse-online.de/de/. Zugegriffen: 26. Aug. 2020

[11] https://www.elhks.de/. Zugegriffen: 26. Aug. 2020

[12] Schaefer JR (2015). Editorial. Klinikarzt.

[13] https://de.wikipedia.org/wiki/Dr._House. Zugegriffen: 26. Aug. 2020

[14] Schäfer JR (2012) Housemedizin. Wiley-VCH, Weinheim. ISBN: 978 3 527 506392

[15] Witzel K, Koch HJ, Kaminski C (2017). Impact of medical TV shows on preprocedural fear of surgical in-house patients. Eur Surg Res.

[16] Schaefer JR, Jerrentrup A, Neubauer A (2010). Neue Ansätze zur Wissensvermittlung oder: was können wir vom Fernsehen lernen? [New approaches to lecture medicine—or: what can we learn from television?]. Dtsch Med Wochenschr.

[17] Jerrentrup A, Müller T, Neubauer A, Schäfer JR, Kauffeld S, Othmer J (2019). Dr. House: Was wir von Hollywood lernen können. Handbuch Innovative Lehre.

[18] Jerrentrup A, Mueller T, Glowalla U, Herder M, Henrichs N, Neubauer A, Schaefer JR (2018). Teaching medicine with the help of “Dr. House”. Plos One.

[19] https://www.focus.de/gesundheit/arzt-klinik/mein-arzt/tid-12198/(MFT) dr-house-medizin-als-krimi_aid_342094.html. Zugegriffen: 26. Aug. 2020

[20] https://www.welt.de/vermischtes/article145416321/Das-ist-der-deutsche-und-echte-Dr-House.html. Zugegriffen: 26. Aug. 2020

[21] https://www.aerzteblatt.de/archiv/76359/Juergen-Schaefer-Der-deutsche-Dr-House. Zugegriffen: 26. Aug. 2020

[22] Dahms K, Sharkova Y, Heitland P, Pankuweit S, Schaefer JR (2014). Cobalt intoxication diagnosed with the help of Dr House. Lancet.

[23] Wise J (2014). TV show House helped doctors spot cobalt poisoning. BMJ.

[24] Schafer JR (2014). Evening News with.

[25] Kolata G (2014) As seen on TV, a medical mystery involving hip implants is solved. https://www.nytimes.com/2014/02/07/health/house-plays-a-role-in-solving-a-medical-mystery.html?_ r=0. Zugegriffen: 26. Aug. 2020

[26] Alter C (2014) TV’s ‘house’ helps solve real-life medical mystery. http://time.com/5375/tvs-house-helps-solve-real-life-medical-mystery/. Zugegriffen: 26. Aug. 2020

[27] https://sciencedirect.altmetric.com/details/2104541. Zugegriffen: 26. Aug. 2020

[28] Allen LA, Ambardekar AV, Devaraj KM, Maleszewski JJ, Wolfel EE (2014). Missing elements of the history. N Engl J Med.

[29] https://www.nejm.org/doi/metrics/10.1056/NEJMcps1213196. Zugegriffen: 26. Aug. 2020

[30] https://www.daserste.de/unterhaltung/quiz-show/hirschhausens-quiz-des-menschen/index.html. Zugegriffen: 26. Aug. 2020

[31] Li W, Watts J, Tan N (2019). From screen to screening: entertainment and news television media effects on cancer screening behaviors. J Health Commun.

[32] Hoffman BL, Cafferty LA, Parul Jain, Shensa A, Rosenthal EL, Primack BA, Sidani JE (2020). Patient-centered communication behaviors on primetime television. J Health Commun.

[33] Heitland L, von Hirschhausen E, Fischer F (2020). Effects of humorous interventions on the willingness to donate organs: a quasi-experimental study in the context of medical cabaret. BMC Public Health.

[34] Betsch C, Küpke NK, Otten L, von Hirschhausen E (2020). Increasing the willingness to participate in organ donation by humorous health communication: experimental evidence. PLoS.

[35] Niemann P, Bittner L, Hauser C, Schrögel P (2020). Science-Slam: Multidisziplinäre Perspektiven auf eine populäre Form der Wissenschaftskommunikation.

[36] https://de.statista.com/statistik/daten/studie/2913/umfrage/fernsehkonsum-der-deutschen-in-minuten-nach-altersgruppen/. Zugegriffen: 26. Aug. 2020

[37] https://www.zdf.de/gesellschaft/menschen-das-magazin/menschen---das-magazin-vom-1-juli-2017-100.html. Zugegriffen: 26. Aug. 2020

[38] https://raul.de/. Zugegriffen: 26. Aug. 2020

[39] https://www.bijan-kaffenberger.de/. Zugegriffen: 26. Aug. 2020

[40] https://www.youtube.com/watch?v=bnmsUeQfzzQ. Zugegriffen: 26. Aug. 2020

[41] Soufi M, Ruppert V, Rinné S (2018). Increased KCNJ18 promoter activity as a mechanism in atypical normokalemic periodic paralysis. Neurol Genet.

[42] https://www.se-atlas.de/. Zugegriffen: 26. Aug. 2020

[43] https://www.uni-bielefeld.de/gesundhw/ag6/downloads/Ergebnisbericht_HLS-GER.pdf. Zugegriffen: 26. Aug. 2020

[44] Hurrelmann K, Schmidt-Kaehler S, von Hirschhausen E, Betsch C, Schaeffer D (2019) Strategiepapier #3 zu den Empfehlungen des Nationalen Aktionsplans. Den Umgang mit Gesundheitsinformationen in den Medien erleichtern Berlin: Nationaler Aktionsplan Gesundheitskompetenz 2019. https://pub.uni-bielefeld.de/record/2933465. Zugegriffen: 26. Aug. 2020

[45] McNeilly DP, Wengel SP (2001). The “ER” seminar: teaching Psychotherapeutic techniques to medical students. Acad Psychiatry.

[46] Pavlov A, Dahlquist GE (2010). Teaching communication and professionalism using a popular medical drama. Fam Med.

[47] http://scienceandentertainmentexchange.org. Zugegriffen: 26. Aug. 2020

[48] https://hollywoodhealthandsociety.org. Zugegriffen: 26. Aug. 2020

[49] https://imblickpunkt.grimme-institut.de/das-bild-von-menschen-mit-behinderungen-in-den-medien/. Zugegriffen: 26. Aug. 2020

